# Children’s Behavioral Development in Correlation with Postpartum Mental Health During Pandemic Period

**DOI:** 10.3390/children13040467

**Published:** 2026-03-28

**Authors:** Arianna Capocasale, Luca Liberati, Danilo Buonsenso, Giulia Bersani, Michela Caprarelli, Daniela Pia Rosaria Chieffo, Ilaria Contaldo, Daniele Gemin, Giulia Giugno, Rosanna Mastricci, Ida Turrini, Chiara Veredice, Ilaria Lazzareschi

**Affiliations:** 1 Medicine and Surgery, Università Cattolica del Sacro Cuore, 00168 Rome, Italy; 2 Pediatric Department, Buzzi Children’s Hospital, 20154 Milan, Italy; 3 Clinical Psychology Unit, Health Management, Fondazione Policlinico Universitario Agostino Gemelli IRCCS, Largo Agostino Gemelli 1, 00168 Rome, Italy; luca.liberati@guest.policlinicogemelli.it (L.L.);; 4 Pediatria, Dipartimento Scienze della Salute della Sonna e del Bambino, Fondazione Policlinico Universitario A. Gemelli IRCSS—Roma, 00168 Rome, Italyilaria.lazzareschi@unicatt.it (I.L.); 5 Neuropsichiatria Infantile, Dipartimento Scienza della Salute della Donna e del Bambino, Fondazione Policlinico A. Gemelli IRCCS, 00168 Rome, Italy; 6 Department of Statistical Sciences, Sapienza University of Rome, 00185 Rome, Italy

**Keywords:** socioemotional development, preschool children, child behavior checklist, postpartum depression, maternal mood, pandemic, behavioral outcomes, pediatric awareness

## Abstract

**Highlights:**

**What are the main findings?**

- Preschoolers assessed in the during-pandemic cohort showed a higher prevalence of non-normal internalizing scores than those assessed in the post-pandemic cohort.
- Maternal SARS-CoV-2 infection at delivery was not associated with children’s CBCL 1½–5 outcomes in this sample.

**What is the implication of the main findings?**

- The findings suggest partial emotional–behavioral recovery following pandemic-related stress exposure, even though age bias should be considered.
- This may help pediatricians identify unusual behaviors in children born during the pandemic and consider early supportive interventions.

**Abstract:**

**Background/Objectives**: Maternal postpartum depressive symptoms and the COVID-19 pandemic have both been identified as potential risk factors for socioemotional difficulties in children. This study aimed to assess behavioral outcomes in young children born to mothers previously screened for postpartum depressive symptoms, comparing cohorts evaluated during and after the pandemic using the Child Behavior Checklist (CBCL 1½–5). **Methods:** An observational follow-up cohort study was conducted on 52 mother–child dyads derived from a previously established maternal cohort screened with the Edinburgh Postnatal Depression Scale (EPDS). Two cohorts were defined according to the child’s birth period: during-pandemic (January–April 2022) and post-pandemic (October–November 2023) groups. Behavioral outcomes were assessed using CBCL 1½–5. Group differences were tested using parametric or non-parametric methods for continuous variables and χ2 or Fisher’s exact tests for categorical variables. Exploratory regression models and sensitivity analyses were also performed. **Results:** Children assessed in the post-pandemic cohort showed a lower prevalence of non-normal internalizing scores than those assessed in the during-pandemic cohort, whereas externalizing outcomes and Total Problems did not significantly differ between groups. In exploratory models, a child’s age showed a near-significant association with internalizing outcomes, suggesting that developmental stage at assessment may have contributed to the observed cohort difference. Maternal SARS-CoV-2 infection at delivery was not associated with children’s behavioral outcomes. **Conclusions:** These findings suggest a possible difference in internalizing behavioral profiles between children assessed in during-pandemic and post-pandemic cohorts. However, this pattern should be interpreted cautiously because the cohorts differed substantially in age at follow-up, and age-related factors may have affected symptom detectability. Continued longitudinal follow-up will be important to clarify whether the observed differences persist over time.

## 1. Introduction

Early childhood, particularly the first 1000 days of life in children, is a period of heightened neuroplasticity during which multiple environmental factors can shape socio-emotional development [1,2]. Among these factors, maternal postpartum depressive symptoms represent one of the most influential determinants of early socioemotional and behavioral trajectories. When caregiving is emotionally available and contingent, mother–infant interactions support the development of adaptive self-regulation and resilience; conversely, early and repeated exposure to stressors may increase vulnerability to later mental health difficulties [3]. Importantly, contemporary models emphasize that these effects are often mediated by dyadic emotion regulation processes—such as affective attunement and multilevel synchrony—through which infants learn to organize emotional arousal and emerging behavioral responses within the caregiver relationship [4].

Among recent global stressors, the COVID-19 pandemic has substantially altered the psychological environment of families with young children. Although initially framed as a public health emergency, the prolonged restrictions and social disruption also constituted a major psychosocial stressor, associated with heightened stress, social isolation, and changes in caregiving routines [4]. During this challenging period, parents—especially mothers in the postpartum phase—reported increased psychological distress [5]. Several studies have suggested that the pandemic’s context, rather than infection status alone, was associated with changes in caregiver well-being and children’s socioemotional adjustment [6,7]. In particular, the loss of social support networks and the closure of early childhood services may have disrupted typical developmental experiences in early childhood, amplifying daily stressors and reducing opportunities for stimulation and social learning during a critical phase of childhood [8].

Evidence from different countries has further highlighted how pandemic-related disruptions affected both parental well-being and children’s behavioral adjustment. A national survey conducted in the United States reported that many parents perceived a deterioration in their own mental health as well as in their children’s behavioral health since the beginning of the pandemic, suggesting that family stress represented a major pathway through which the crisis affected children’s emotional functioning [9]. Similarly, research conducted in Italy during the first wave of the pandemic found that higher levels of parental stress were significantly associated with increased emotional and behavioral problems in children, emphasizing the central role of the caregiving environment in shaping children’s psychological responses to the crisis [10]. Consistent findings have also been reported in Spain, where children exposed to strict lockdown measures showed higher emotional and behavioral difficulties, particularly in families where parents experienced elevated psychological distress and difficulties in coping with confinement-related stressors [11]. Together, these studies highlight the importance of considering family functioning and parental mental health as key contextual factors influencing children’s socioemotional development during large-scale crises.

In addition to its direct impact on children’s development, reported in the literature, data collected at our institution (Fondazione Policlinico Universitario Agostino Gemelli IRCCS, Rome) during the pandemic indicated that mothers who delivered between early 2020 and 2022 showed significantly higher scores on the Edinburgh Postnatal Depression Scale (EPDS) compared to those who delivered afterward, consistent with international findings [12,13,14]. Postpartum depressive symptoms may affect child development not only through symptom severity per se, but also through their impact on dyadic processes—including reduced maternal sensitivity, responsiveness, and affective synchrony—which can constrain the infant’s opportunities to develop secure attachment representations and early regulatory skills [15]. This dyadic perspective highlights that risk is often expressed through disruptions in parent–child co-regulation, rather than through maternal symptoms in isolation [16].

Building on these premises, this study aimed to examine how children’s socioemotional and behavioral development is associated with exposure to two potential risk factors for behavioral alteration in offspring, which in our sample occurred simultaneously: maternal postpartum depressive symptoms and the COVID-19 pandemic context. This study was conceived as a continuation of the previous Gemelli project, which focused on maternal mood variations during the pandemic. Here, we extend the investigation to the children of those same mothers, using the Child Behavior Checklist (CBCL 1½–5) to assess socioemotional development. Given the complex interplay between maternal symptoms, dyadic regulation, and contextual stress, our analyses were designed to explore associations while acknowledging key sources of heterogeneity (e.g., developmental stage at assessment and follow-up participation). Accordingly, the interpretation of cohort differences was framed cautiously, considering that developmental timing and measurement sensitivity may influence observed internalizing profiles.

## 2. Materials and Methods

### 2.1. Study Design

This observational follow-up cohort study based on a previously established maternal cohort was conducted at the Unit of General Pediatrics of Fondazione Policlinico Universitario A. Gemelli IRCCS (Rome, Italy). It represents the second phase of a longitudinal research project initiated three years earlier.

The present phase aimed to evaluate the behavioral development of children born to mothers who had previously participated in the first phase of the project and who delivered either during the pandemic period (January–April 2022) or in a later period defined as post-pandemic (October–November 2023), after the end of the WHO-declared global health emergency and after the lifting of the major restrictive measures affecting family and social life in Italy.

### 2.2. Participants

The study sample was derived from a previously established maternal cohort screened for postpartum depressive symptoms using the Edinburgh Postnatal Depression Scale (EPDS). Mothers were originally recruited as part of a project aimed at identifying postpartum depressive symptoms in women with or without SARS-CoV-2 infection at the time of delivery.

For the present follow-up phase, all 144 mother–child dyads from the original cohort were recontacted and invited to participate in the study. Of these, 52 mothers returned a completed follow-up questionnaire and were, therefore, included in the final analytical sample.

Participants belonged to two birth cohorts defined according to the child’s date of birth:

- During-pandemic cohort: children born between January and April 2022;
- Post-pandemic cohort: children born between October and November 2023.

The rationale for this follow-up was based on the findings of the first project, which suggested that postpartum depressive symptoms were associated with the broader pandemic context rather than with SARS-CoV-2 infection itself. Therefore, the present study aimed to investigate whether exposure to the pandemic context during early life might also be associated with differences in children’s socioemotional development.

Inclusion criteria were participation in the original maternal cohort screened with EPDS, birth of the child within one of the predefined time periods, and completion of the follow-up assessment. No additional exclusion criteria were applied beyond the predefined cohort selection and availability of completed follow-up questionnaires.

### 2.3. Measures and Instruments

#### 2.3.1. Child Behavior Checklist 1½–5 (CBCL)

The CBCL 1½–5 is a standardized questionnaire of the ASEBA system (Achenbach System of Empirically Based Assessment) administered to mothers with the aim of evaluating their child’s behavioral and psychological profile, aged between 18 months and 5 years.

The questionnaire consists of 99 items, each rated by the caregiver on a three-point Likert scale (where 0 = not true, 1 = somewhat or sometimes true, and 2 = very true or often true).

Raw scores are converted into norm-referenced T-scores using the standardized scoring procedures described in the ASEBA manual, allowing comparison with age-normalized reference populations [17].

T-scores were organized into several broadband domains:

- Internalizing scale: including behaviors like social withdrawal, anxiety and depressive symptoms, which tend to be internalized and less visible.
- Externalizing scale: including problems such as disobedience, impulsiveness and hyperactivity which tend to manifest more openly in the social context.
- Total Problem scale: representing a global index of emotional and behavioral problems.
- Other Problems scale: capturing additional behavioral issues not included in the main scales.

In accordance with ASEBA administration guidelines, the CBCL is designed to be completed independently by caregivers who know their child well. For this reason, the questionnaire was sent electronically to the mothers, who completed it autonomously and returned it via email. This procedure preserves the self-report nature of the instrument and minimizes potential interviewer influence on parental responses [17].

#### 2.3.2. Supplementary Questionnaire

In addition to the CBCL, an ad hoc supplementary questionnaire was created using Google Forms in order to collect contextual information regarding the child’s environment and potential factors that might have influenced development beyond the primary variables of interest. This tool included questions related to:

- Traumatic or stressful events that occurred in the previous 12 months;
- Changes in household composition or in place of residence;
- Other eventual subjective factors considered relevant by mothers for their child’s well-being;
- Demographic data.

### 2.4. Procedure and Data Collection

Data collection was conducted remotely. Mothers received the CBCL questionnaire via email and were asked to complete it independently and return the document in digital form (scanned or electronically completed).

Each child’s CBCL results were linked to the corresponding maternal EPDS data collected during the first phase of the study through anonymized unique identifiers generated during the original cohort enrollment.

Demographic and clinical variables collected included maternal age, maternal education level, maternal EPDS score, return to work after childbirth, maternal SARS-CoV-2 infection at delivery, the child’s age at follow-up assessment, parity, maternal nationality, gestational age at birth, birth weight, and breastfeeding modality.

### 2.5. Statistical Analysis

Statistical analyses were performed using the open-source software R-studio (RS). CBCL T-scores were originally categorized as normal, borderline, or clinical according to ASEBA normative thresholds. For inferential analyses, the internalizing scale was modeled using ordinal categories (0/1/2), preserving the ordered structure of the outcome. In contrast, externalizing outcomes and Total Problems were analyzed as binary outcomes due to the limited number of observations in the clinical range. This approach was adopted to ensure model stability and avoid unreliable estimates associated with sparse outcome categories. Continuous variables were tested for normality using the Shapiro–Wilk test. Depending on data distribution, group comparisons were conducted with parametric (*t*-test) or non-parametric (Mann–Whitney U) tests for continuous variables, and χ^2^ or Fisher’s exact tests for categorical variables. Correlation analyses were used to explore associations between maternal EPDS scores and children’s CBCL T-scores. Sensitivity analyses were conducted to assess the robustness of the results, with statistical significance set at *p* < 0.05.

### 2.6. Ethical Consideration

The study was conducted in accordance with the ethical standards of the Declaration of Helsinki and approved by the Ethics Committee of Fondazione Policlinico Universitario A. Gemelli IRCCS (approval code 0031589/22, date 6 October 2022).

Written informed consent was obtained digitally from all participants before inclusion in the study. Data was anonymized prior to analysis, and no identifying information was collected or stored.

### 2.7. Data Availability Statement

The dataset generated and analyzed during the current study is stored in a secure institutional repository at Fondazione Policlinico Universitario A. Gemelli IRCCS. Data will be made available from the corresponding author upon reasonable request.

## 3. Results

### 3.1. Sample

Fifty-two mother-child dyads were assessed (with a follow-up participation rate of 36.1%). Children belonged to two birth cohorts: during-pandemic (January–April 2022, n = 30) and post-pandemic cohorts (October–November 2023, n = 22). Follow-up CBCL assessments were conducted between March and May 2025. Statistical analyses were conducted using RStudio (version 4.4.3 open source).

#### 3.1.1. Demographic Characteristics

**Figure 1:**
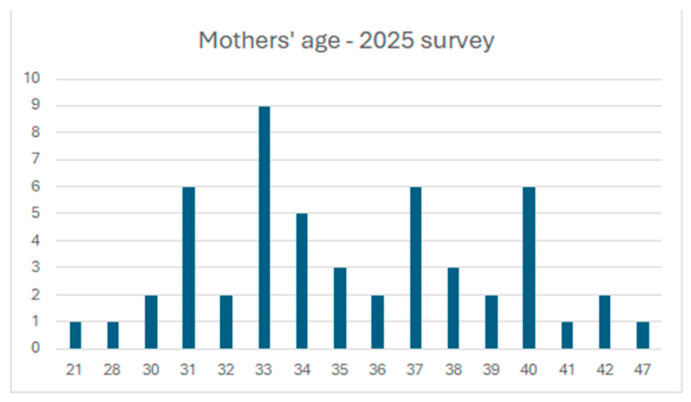
Distribution of maternal age in the follow-up sample (2025 follow-up survey). The histogram shows the frequency of mothers by age at the time of follow-up participation.

Maternal age had a mean of 35.2 years (median 34.5 years) in the follow-up sample, with the most frequent age being 33 years. Only one participant was under 18 years of age at delivery in the original cohort. The distribution of maternal age within the follow-up sample is illustrated in Figure 1.

**Figure 2:**
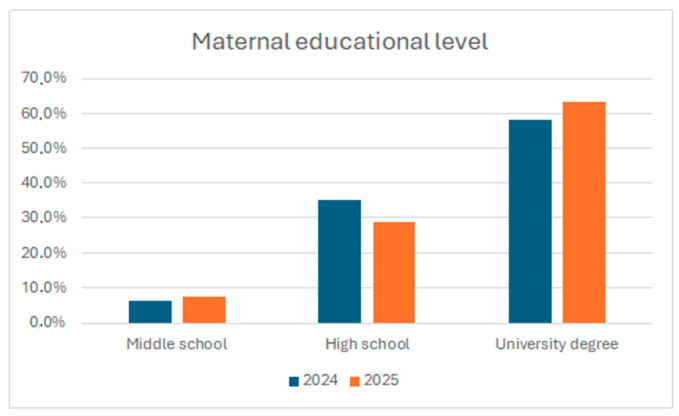
Distribution of maternal educational level in the original cohort (2024) and in the follow-up sample (2025). Educational attainment is shown as the proportion of mothers with primary school, high school, or university degree education in the two survey phases.

Regarding educational level, 48 out of 52 mothers (92%) completed education beyond middle school, indicating a predominantly medium-to-high educational background. The distribution of maternal educational level in the original cohort and in the follow-up sample is presented in Figure 2.

**Figure 3:**
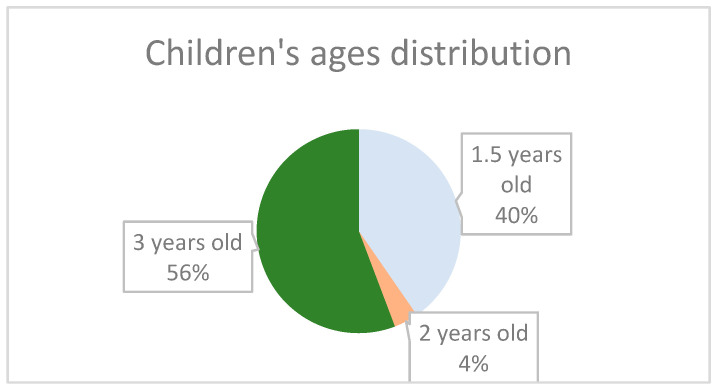
Distribution of children’s age at follow-up according to birth cohort. Children born during the pandemic were assessed at approximately 3 years of age, whereas children in the post-pandemic cohort were assessed at approximately 18 months, corresponding to the lower boundary of the CBCL 1½–5 age range.

Children’s age at follow-up differed between cohorts by design. Children born during the pandemic were approximately 3 years old, while those born in the later cohort were approximately 18 months old, corresponding to the lower boundary of the CBCL 1½–5 assessment range. The distribution of children’s age at follow-up in the two cohorts is illustrated in Figure 3.

**Figure 4:**
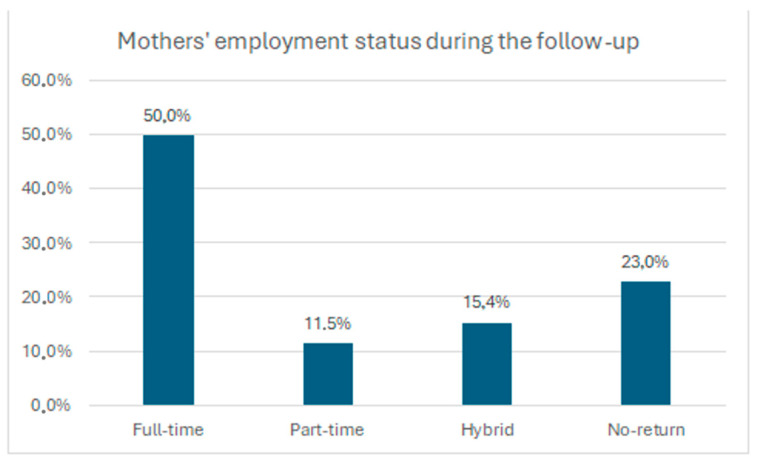
Maternal employment status at the time of follow-up. Distribution of mothers according to employment status, including full-time work, part-time work, hybrid work arrangements, and no return to work after childbirth.

With regard to maternal employment status at the time of follow-up, 50% of mothers had returned to full-time work, while others reported part-time (11.5%), hybrid (15.4%), or no return to work (23%). These differences reflect the heterogeneity of postpartum work arrangements within the sample. The distribution of maternal employment status at follow-up is illustrated in Figure 4.

#### 3.1.2. Attrition Rate

Of the 144 mother–child dyads originally enrolled in the first phase of the study, 52 participated in the follow-up assessment, corresponding to a follow-up rate of 36.1% and an attrition rate of 63.9%.

### 3.2. Maternal Mood (EPDS)

The mothers included in the present study had been previously screened for postpartum depressive symptoms using the Edinburgh Postnatal Depression Scale (EPDS) during the first phase of the project.

Among the 52 mothers who participated in the follow-up assessment, the mean EPDS score at baseline was 7.3, and 13 mothers (25%) had an EPDS score >10.

Among the 92 mothers who did not participate in the follow-up phase, the mean baseline EPDS score was 5.5, and 12 (13%) had an EPDS score >10.

These findings indicate that mothers who participated in the follow-up tended to have slightly higher EPDS scores at baseline compared with non-responders.

**Figure 5:**
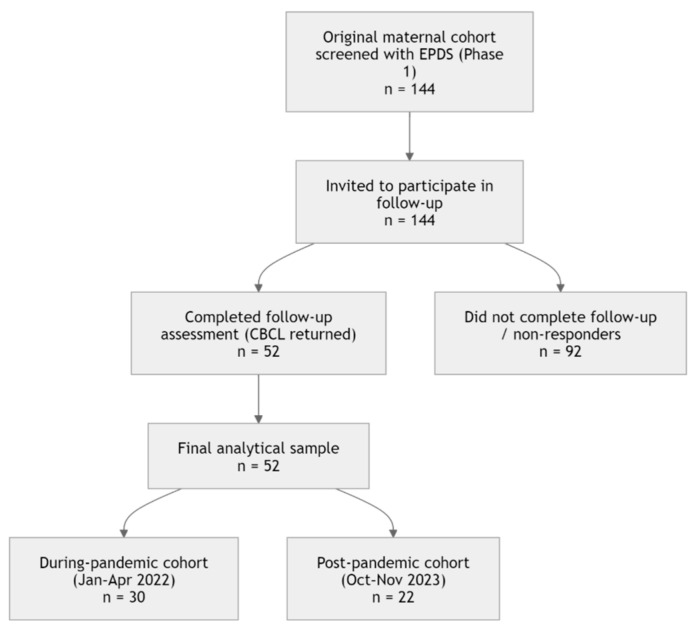
Flow diagram of participant selection from the original maternal cohort to the final analytical sample included in the follow-up study.

The distribution of baseline EPDS scores among mothers included in the follow-up sample is illustrated in Figure 5.

### 3.3. Child Socioemotional Outcomes (CBCL 1½–5)

Across the whole children sample, there was a clear difference in child results between the two cohorts, as summarized in Table 1:

- During Pandemic (3 years, n = 30):
  ○ Internalizing scale: 21 normal (70.0%), 6 borderline (20.0%), and 3 clinical (10.0%);
  ○ Externalizing scale: 23 normal (76.7%), 7 borderline (23.3%), and 0 clinical;
  ○ Total Problems: 25 normal (83.3%), 5 borderline (16.7%), and 0 clinical;
  ○ Other Problems (raw): mean = 7.50.
- Post-pandemic (18 months, n = 22):
  ○ Internalizing scale: 21 normal (95.5%), 1 borderline (4.5%), and 0 clinical;
  ○ Externalizing scale: 21 normal (95.5%), 1 borderline (4.5%), and 0 clinical;
  ○ Total Problems: 19 normal (86.4%), 3 borderline (13.6%), and 0 clinical;
  ○ Other Problems (raw): mean = 5.19.

#### Comparisons Between Cohorts (Primary Contrast)

- Internalizing (non-normal vs normal) score: 9/30 vs. 1/22 → Fisher’s exact *p* = 0.032, OR = 9.00 (a non-normal score was more likely for the during-pandemic cohort).
- Externalizing (non-normal vs normal) score: 7/30 vs. 1/22 → *p* = 0.118, OR = 6.39 (ns).
- Total Problems (non-normal vs normal): 5/30 vs. 3/22 → *p* = 1.000, OR = 1.27 (ns).

Overall, internalizing symptoms were more frequent in children assessed during the pandemic, whereas the distribution of externalizing outcomes and Total Problems was comparable between cohorts.

**Figure 6:**
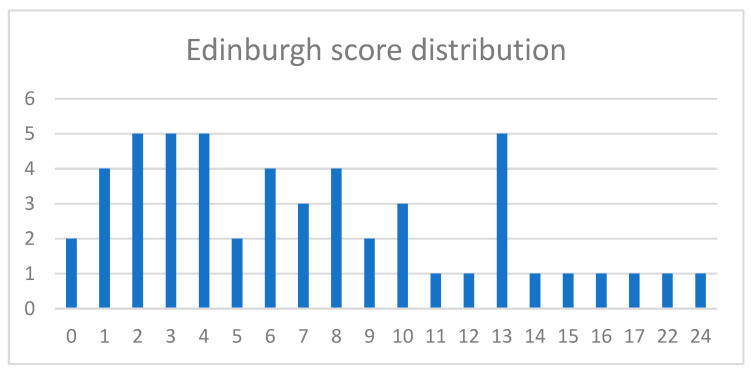
Distribution of baseline Edinburgh Postnatal Depression Scale (EPDS) scores among mothers who participated in the follow-up phase. Bars represent the frequency of EPDS scores recorded during the first phase of the study.

As shown in Figure 6, the prevalence of internalizing problems was significantly higher in children assessed during the pandemic, whereas externalizing outcomes and Total Problems did not differ between cohorts.

### 3.4. Exploratory Models and Sensitivity Analyses

Ordinal logistic models (internalizing scale 0/1/2) and binary logistic models (externalizing scale 0/1; Total Problems 0/1) were estimated. The choice of the models reflected the structure of the outcomes. The internalizing scale retained three ordered categories (normal, borderline, and clinical) and was therefore analyzed using ordinal logistic regression, which models cumulative probabilities across ordered outcome levels. In contrast, the externalizing scale and Total Problems were analyzed as binary outcomes. In these cases, logistic regression provides an appropriate framework because it models the probability of the event while constraining predicted probabilities within the interval [0, 1]. For internalizing outcomes, maternal EPDS scores were not significantly associated with child outcomes (β = −0.032; *p* = 0.635). Child age showed a near-significant effect (β = 1.417; *p* = 0.053), suggesting higher odds of internalizing difficulties at 3 years compared with 18 months. SARS-CoV-2 infection at delivery showed no significant association (β = 0.53; *p* = 0.451). Scores for “Other Problems” showed a positive trend-level association (β = 0.252; *p* = 0.068).

For externalizing outcomes, no predictors reached statistical significance in univariable models. In multivariable models, child age approached significance (β = 0.921; *p* = 0.134; OR ≈ 2.51 per additional year), while maternal age and COVID status at delivery were not associated with the outcome. Scores for “Other Problems” were significantly associated with externalizing outcomes (β = 0.22; *p* = 0.014; OR ≈ 1.25 per point).

For Total Problems, scores for “Other Problems” were significantly associated with the outcome (β = 0.3253; *p* = 0.004; OR ≈ 1.38 per point), while no other predictors reached statistical significance.

**Figure 7:**
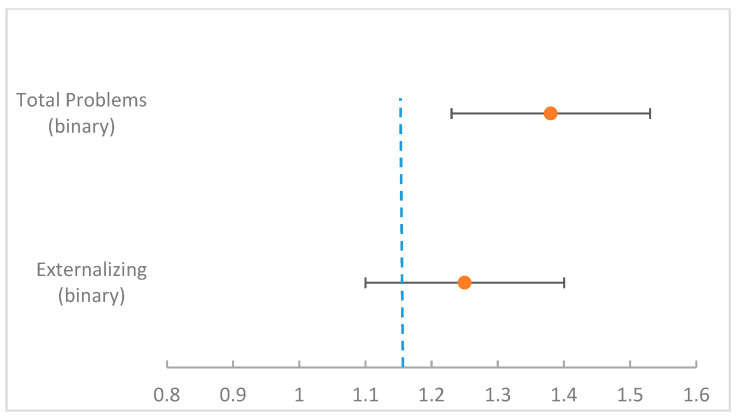
Association between scores for “Other Problems” and CBCL outcomes. Forest plot of Odds Ratios (ORs) derived from logistic regression models examining the association between raw scores for “Other Problems” and CBCL outcomes. Each point represents the estimated OR (β converted to OR) adjusted for child age. Error bars represent 95% confidence intervals, indicating the precision of the estimates and their statistical significance.

These results, which are summarized in Table 2 and illustrated in Figure 7, are consistent with non-parametric sensitivity checks and alternative coding strategies for covariates. Given the limited sample size (n = 52), more complex model specifications were intentionally avoided in order to reduce the risk of overfitting.

**Figure 8:**
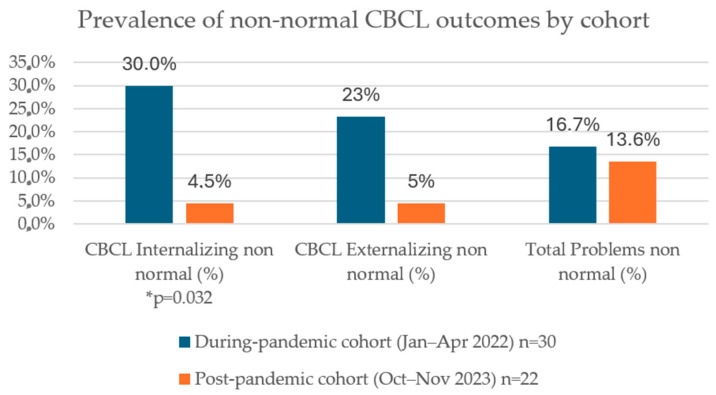
Prevalence of non-normal CBCL outcomes by cohort: proportion of children showing borderline or clinical CBCL scores in internalizing, externalizing, and Total Problems scales during and after the COVID-19 pandemic. Significant between-group differences were observed only for the internalizing scale (* *p* = 0.032, Fisher’s exact test).

Figure 8 summarizes the logistic regression models, highlighting a significant positive association between scores for “Other Problems” and both externalizing and Total Problems outcomes.

## 4. Discussion

For the purpose of this study, we followed a cohort of children born to mothers previously screened for postpartum depressive symptoms with EPDS during and after the COVID-19 pandemic era in order to explore whether children’s socioemotional profiles differed between the two birth cohorts.

Three main findings emerged. First, internalizing difficulties were more frequent in children assessed in the during-pandemic cohort compared with those assessed in the post-pandemic cohort, whereas externalizing outcomes and Total Problems were broadly similar between groups. However, this difference must be interpreted cautiously because the two cohorts were assessed at different ages (approximately 3 years vs. 18 months), and age-related factors may partially account for the observed differences.

Second, child age showed a consistent trend in regression models, with higher odds of internalizing outcomes at 3 years compared with 18 months. This finding further supports the possibility that visible development of symptoms may have contributed to the contrast between cohorts.

Third, the “Other Problems” raw score—an index capturing atypical or additional caregiver concerns—was positively associated with both externalizing outcomes and Total Problems. By contrast, neither maternal EPDS score nor maternal SARS-CoV-2 infection around delivery was associated with children’s CBCL outcomes in this sample.

### 4.1. Age-Related Interpretation of Cohort Differences

The age difference between the two cohorts represents an important interpretative factor. Children in the during-pandemic cohort were assessed at approximately 3 years of age, whereas children in the post-pandemic cohort were assessed at approximately 18 months, which represents the lower boundary of the CBCL 1½–5 instrument.

Two non-exclusive explanations should therefore be considered.

First, there is developmental visibility: at around 3 years of age, internalizing behaviors such as separation anxiety, social inhibition, and withdrawal are typically more recognizable by caregivers. Consequently, caregivers may be more likely to report subtle internalizing symptoms at this age compared with the toddler stage.

Second, there is instrument sensitivity: when behavioral screening tools are applied at the lower boundary of their validated age range, some emotional and behavioral problems may be less easily detected because certain behaviors have not yet fully emerged developmentally. The ASEBA manual itself notes that some behavioral manifestations become more clearly identifiable only at later developmental stages [17].

Taken together, these considerations suggest that the observed difference between the two cohorts could reflect a combination of contextual factors related to the pandemic period and age-related detectability of behavioral symptoms.

Furthermore, these observations lay the foundation for an important reflection on the importance of a future hypothetical reassessment of our sample in a school-age cohort, when internalizing outcomes and executive-function-related behaviors stabilize and instruments show better discrimination.

### 4.2. Interpreting Cohort Differences: Pandemic Versus Infection

Despite the age-related considerations discussed above, the higher proportion of internalizing problems observed in the during-pandemic cohort remains consistent with the hypothesis that the pandemic functioned as a psychosocial stressor affecting early developmental environments.

The COVID-19 pandemic profoundly altered family routines, access to social support networks, and early socialization opportunities for young children. Several studies have reported increased emotional and behavioral vulnerability among preschool children during the pandemic period, suggesting that broader contextual stressors may have played a significant role in shaping early developmental trajectories [18].

Importantly, in our dataset, maternal SARS-CoV-2 infection around delivery was not associated with child behavioral outcomes. This observation supports the interpretation that the broader psychosocial context of the pandemic, rather than the biological infection itself, may have been more relevant for early socioemotional development.

Nevertheless, given the age imbalance between cohorts and the observational design of this study, these findings should be interpreted as suggestive rather than causal evidence of a pandemic-period effect.

### 4.3. Attrition Rate and Sample Demographic Characteristics

Another important aspect concerns the attrition rate observed between the two phases of the study. Of the original 144 mother–child dyads enrolled in the first phase, only 52 participated in the follow-up assessment, corresponding to a follow-up rate of approximately 36%.

Baseline comparisons between responders and non-responders were conducted to evaluate potential selection bias. Mothers who participated in the follow-up were on average slightly older than non-responders, while educational levels were comparable between the two groups, with a high proportion of mothers having completed secondary or higher education in both phases.

Interestingly, responders showed slightly higher baseline EPDS scores compared with non-responders, suggesting that the final sample does not represent a lower-risk subgroup of the original cohort. This pattern may reflect a participation bias whereby mothers experiencing greater psychological distress were more motivated to remain engaged in the study.

Such selection processes should be considered when interpreting the findings, as they may influence the representativeness of the final sample.

### 4.4. Maternal Mood and Child Behavior: Level Versus Context

Within the maternal cohort included in this study, postpartum depressive symptoms measured with EPDS were not significantly associated with children’s CBCL outcomes.

However, this finding should be interpreted cautiously. The EPDS was administered at a single time point during the postpartum period and, therefore, captures a snapshot of maternal emotional state rather than its longitudinal course. Several studies have shown that postpartum depressive symptoms may fluctuate substantially across the first year after childbirth, highlighting the importance of repeated assessments to capture symptom chronicity and developmental timing effects [19].

Moreover, screening tools such as the EPDS do not directly measure the quality of mother–child interactions or caregiving behaviors that may mediate the relationship between maternal mood and child development. Consequently, the absence of an association in this study should not be interpreted as evidence that maternal mental health is irrelevant for child socioemotional development, but rather as a limitation of the available measurement design.

### 4.5. Interpreting the “Other Problems” Scale

Across the regression models, higher raw scores for “Other Problems” were associated with greater odds of both externalizing outcomes and Total Problems (OR ≈ 1.25 and 1.38 per point, respectively) and showed a borderline association with internalizing outcomes.

Although the “Other Problems” scale is not a formal syndrome scale within the CBCL framework, it includes caregiver-reported concerns (e.g., sleep disruption, regressions, and somatic complaints) that are not captured within the main syndrome groupings but may still reflect relevant aspects of child adjustment [18].

Clinically, this underscores the value of listening to non-syndromic concerns in healthy child visits: they may flag children who would benefit from brief, supportive interventions (parent guidance on routines, emotion coaching, and sleep hygiene) even when syndrome T-scores are not yet elevated.

At the same time, because this category is derived from the same CBCL instrument used to measure the main behavioral domains, some degree of conceptual or measurement overlap with other scales cannot be excluded. For this reason, the association observed in our analyses should be interpreted cautiously and may reflect a broader signal of caregiver-perceived developmental concerns rather than a distinct behavioral construct.

### 4.6. Strengths and Limitations

Strengths include: (i) a clinically relevant sampling frame; (ii) standardized outcomes (CBCL 1½–5) with normative categorization; and (iii) analyses that separated period effects from infection status, allowing a cleaner interpretation.

Several limitations should nevertheless be considered.

First, the relatively small follow-up sample and the substantial attrition rate may have introduced selection bias.

Second, the age imbalance between cohorts (3 years vs. 18 months) represents a major methodological limitation, as developmental stage may influence both the visibility of internalizing symptoms and the sensitivity of the CBCL instrument.

Third, behavioral outcomes were assessed using a single informant (maternal report) at a single time point. Multi-informant assessments, including teachers or childcare providers, would strengthen construct validity.

Finally, maternal mood was measured using a single EPDS assessment, which does not capture symptom chronicity or timing relative to critical developmental windows.

### 4.7. Clinical Implications

Despite these limitations, several clinically relevant considerations emerge.

1. Context matters: Children assessed during the pandemic showed more internalizing difficulties, consistent with the stress-laden caregiving surroundings. Thus, pediatricians should inquire proactively about routines, sleep, separations and emotion regulation in children born or cared for during high-stress periods.
2. Attend to “small signals”: Elevated scores for “Other Problems”—even with otherwise “normal” scales—might prompt anticipatory guidance and early support (sleep/routine interventions, parent–child play-based strategies, and brief parenting programs), for symptoms which are low-risk and potentially protective.

Nevertheless, the exploratory nature of the present study and the modest sample size warrant cautious interpretation of these clinical implications.

### 4.8. Future Directions

Three design refinements follow directly from our findings:

1. Longitudinal follow-up is needed in school-age cohorts, with repeated CBCL and executive-function measures, to determine whether the internalizing difference during the pandemic persists, normalizes, or shifts profile.
2. Richer exposure modeling is required with repeated EPDS to index timing and chronicity, plus micro-level caregiving indices (sensitivity, responsiveness) to test mediation from maternal mood to child adjustment.
3. Multi-informant, or multi-method assessments (mother, teacher/daycare; clinical observational) and digital data capture (online ASEBA scoring) could reduce burden and attrition. Pre-registration and targeted sample-size planning would increase power for small effects.

## 5. Conclusions

The present study suggests that children assessed in the cohort born during the COVID-19 pandemic showed a higher prevalence of internalizing behavioral difficulties compared with those assessed in the post-pandemic cohort. However, this difference should be interpreted cautiously, as the two cohorts were assessed at different developmental stages (approximately 3 years vs. 18 months), and age-related factors may partially explain the observed pattern.

Furthermore, our findings indicate that children’s behavioral outcomes were not associated with maternal SARS-CoV-2 infection around delivery, supporting the hypothesis that broader contextual stress related to the pandemic environment may have played a more relevant role.

At the same time, the absence of a significant association between maternal EPDS scores and child outcomes should be interpreted carefully, given that EPDS captures postpartum depressive symptoms at a single time point and does not reflect their evolution over time.

From a clinical perspective, these findings highlight the importance of maintaining careful developmental monitoring of children exposed to periods of widespread societal stress. Early identification of subtle internalizing symptoms or atypical emotional responses may allow timely supportive interventions to take place and promote healthier developmental trajectories.

Future longitudinal follow-up of this cohort into school age will be essential to determine whether the patterns observed in early childhood persist, attenuate, or evolve across development.

## Figures and Tables

**Table 1:** Sample characteristics and CBCL outcomes by cohort.

Characteristic	During-Pandemic Cohort (January–April 2022) n = 30	Post-Pandemic Cohort (October–November 2023) n = 22
Child’s age at assessment	3 years	18 months
Maternal EPDS, mean	8.9	6.8
EPDS > 9, n (%)	36.7%	27.3%
CBCL Internalizing, (%)—Normal	70.0%	95.5%
CBCL Internalizing, (%)—Borderline	20.0%	4.5%
CBCL Internalizing, (%)—Clinical	10.0%	0%
CBCL Externalizing, n (%)—Normal	76.7%	95.5%
CBCL Externalizing, n (%)—Borderline	23.3%	4.5%
CBCL Externalizing, n (%)—Clinical	0%	0%
CBCL Total Problems, n (%)—Normal	83.3%	86.4%
CBCL Total Problems, n (%)—Borderline	16.7%	13.6%
CBCL Total Problems, n (%)—Clinical	0%	0%
Other Problems (raw), mean	7.50	5.19

Notes: CBCL 1½–5 T-scores were converted to normative categories (0 = normal; 1 = borderline; 2 = clinical) using age/sex-specific norms. “Other Problems” is reported as the raw mean as it was scored continuously in the dataset.

**Table 2:** Key regression results (parsimonious models).

Outcome	Predictor(s)	β (SE)	*p*-Value	Interpretation
Internalizing (ordinal)	EPDS (raw)	−0.032	0.635	ns
	Child age (3 y vs. 1.5 y)	1.417	0.053	trend ↑ risk at 3 y
	Other Problems (raw)	0.252	0.068	trend
Externalizing (binary)	Other Problems (raw)	0.220	0.014	OR ≈ 1.25 per point
	Child age (years)	0.921	0.134	trend; OR ≈ 2.51 per year
Total Problems (binary)	Other Problems (raw)	0.3253	0.004	OR ≈ 1.38 per point

Notes: β, from ordinal (internalizing scale) or binary logistic (externalizing scale and Total Problems) regressions; OR, reported when interpretable from β (externalizing/Total).

## Data Availability

The datasets generated and analyzed during the current study are stored at Fondazione Policlinico Universitario A. Gemelli IRCCS, Rome, Italy, and are available from the corresponding author on reasonable request, in accordance with institutional data protection and ethical guidelines.

## Abbreviations

The following abbreviations are used in this manuscript:

CBCL	Child Behavior Checklist
EPDS	Edinburgh Postnatal Depression Scale
IRB	Institutional Review Board
SD	Standard Deviation
OR	Odds Ratio
